# Transcriptomic Profiling after In Vitro Δ^8^-THC Exposure Shows Cytoskeletal Remodeling in Trauma-Injured NSC-34 Cell Line

**DOI:** 10.3390/ph16091268

**Published:** 2023-09-07

**Authors:** Luigi Chiricosta, Simone D’Angiolini, Agnese Gugliandolo, Stefano Salamone, Federica Pollastro, Emanuela Mazzon

**Affiliations:** 1 IRCCS Centro Neurolesi “Bonino-Pulejo”, Via Provinciale Palermo, Contrada Casazza, 98124 Messina, Italy; 2 Department of Pharmaceutical Sciences, University of Eastern Piedmont, Largo Donegani 2, 28100 Novara, Italy

**Keywords:** neuronal cell death, in vitro, NSC-34, Δ^8^-tetrahydrocannabinol, transcriptomic analysis, cytoskeleton remodeling, JAK/STAT signaling

## Abstract

Neuronal cell death is a physiological process that, when uncontrollable, leads to neurodegenerative disorders like spinal cord injury (SCI). SCI represents one of the major causes of trauma and disabilities worldwide for which no effective pharmacological intervention exists. Herein, we observed the beneficial effects of Δ^8^-Tetrahydrocannabinol (Δ^8^-THC) during neuronal cell death recovery. We cultured NSC-34 motoneuron cell line performing three different experiments. A traumatic scratch injury was caused in two experiments. One of the scratched was pretreated with Δ^8^-THC to observe the role of the cannabinoid following the trauma. An experimental control group was neither scratched nor pretreated. All the experiments underwent RNA-seq analysis. The effects of traumatic injury were observed in scratch against control comparison. Comparison of scratch models with or without pretreatment highlighted how Δ^8^-THC counteracts the traumatic event. Our results shown that Δ^8^-THC triggers the cytoskeletal remodeling probably due to the activation of the Janus Kinase Signal Transducer and Activator of Transcription (JAK/STAT) signaling pathway and the signaling cascade operated by the Mitogen-Activated Protein (MAP) Kinase signaling pathway. In light of this evidence, Δ^8^-THC could be a valid pharmacological approach in the treatment of abnormal neuronal cell death occurring in motoneuron cells.

## 1. Introduction

Neuronal cell death is a physiological process occurring in healthy individuals. Nevertheless, when cell death becomes out of control, it can trigger several neurodegenerative diseases, such as Alzheimer’s disease, Parkinson’s disease or Amyotrophic Lateral Sclerosis [1]. Additionally, neuronal cell death can also be caused by traumatic events such as spinal cord injury (SCI) [2]. The World Health Organization declares that, annually, from 250,000 to 500,000 people fall into SCI [3]. The consequences of this injury, along with other neurodegenerative diseases, are devastating for individuals and their families. It can lead to partial or complete loss of both motor or sensory functions causing profound cognitive and motor impairments, loss of independence and diminished quality of life [4,5,6]. Therefore, understanding the mechanisms underlying neuronal cell death and exploring potential therapeutic interventions are crucial for developing effective treatments [7].

**Figure 1:**
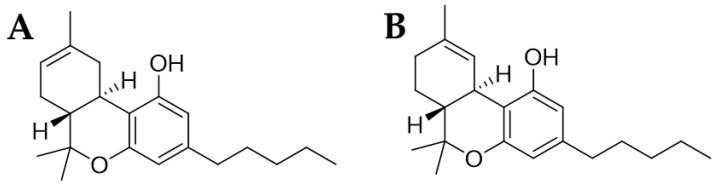
Chemical structure of Δ^8^-THC (**A**) and Δ^9^-THC (**B**).

To date, there is not an effective therapy for SCI. Several strategies were adopted to counteract their consequences, but pharmacological therapies for preventing neuronal cell death are limited [8]. For this reason, research is focused on the discovery of new treatments able to ameliorate the quality of life of the patients with the injury. Recently, research is showing promising results with treatments based on derivatives of cannabinoids [9,10]. Δ^8^-tetrahydrocannabinol (Δ^8^-THC), as well as the other cannabinoids, have emerged as potential candidates for mitigating neuronal cell death due to their neuroprotective properties. Particularly, Δ^8^-THC, whose chemical structure is shown in Figure 1A, is an isomer of Δ^9^-THC (Figure 1B) due to the position of the double chemical bond.

Indeed, Δ^8^-THC exhibits a lower affinity for cannabinoid receptors, resulting in reduced psychoactive effects compared to Δ^9^-THC. For this reason, Δ^8^-THC may represent a safer and more tolerable option for therapeutic applications. Δ^8^-THC is produced by *Cannabis sativa* in very low concentrations but it can be synthetized from cannabidiol (CBD). Δ^8^-THC seems to have analgesic and anti-depressant properties, decrease intra-ocular pressure and mitigate chemotherapy side effects [11,12,13]. Nevertheless, to our knowledge, this molecule was never used in motor-neuron-injured models but cannabinoids already highlighted pain and locomotor function improvement in pre-clinical models [14]. It is noteworthy that no administration protocol exists [15].

Our research group has already studied the effect of Δ^8^-THC in the modulation of different pathways and biological processes in different conditions. In detail, we studied Δ^8^-THC effects in retinoic acid-differentiated SH-SY5Y cell lines. SH-SY5Y are human neuroblastoma cells that, in that experiment, were used to evaluate the potential effects of Δ^8^-THC on the brain. Actually, Δ^8^-THC seems to better influence the activity of glutamatergic and cholinergic synapses [16]. Additionally, this molecule was also able to provide a neuroprotective effect against amyloid beta (Aβ_1–42_), reducing the stress on the endoplasmic reticulum with a consequent drop of neuronal apoptosis [17]. Thus, Δ^8^-THC could effectively protect neuronal cells from death by modulating different cell survival and promoting neuroprotection.

In line with these results, herein, we wanted to test the effects of Δ^8^-THC in a model of scratch injury. Specifically, in this traumatic injury model, performed in vitro, a mechanical injury is caused on the cultured cells [18,19].

Due to the complexity of the scratch injury, we focused our attention on the protective effects of Δ^8^-THC triggered in a population of motor neurons. Specifically, we used the differentiated NSC-34 cell line as a control group and two scratch models. One of the scratch models was treated with Δ^8^-THC. Then, we performed an RNA-seq comparative analysis of the three groups to identify novel signaling pathways activated by Δ^8^-THC in response to the neuronal cell death induced by trauma.

The findings from our study have the potential to provide valuable insights into the molecular mechanisms underlying the effects of Δ^8^-THC to counteract cell death.

## 2. Results

### 2.1. Transcriptomic Profiles Analysis

The efficacy of Δ^8^-THC in the reduction of negative effects caused by axonal damage was tested through the evaluation of the transcriptomic profile of three different groups: control group (CTRL), scratch group (scratch) and scratch group pre-treated with Δ^8^-THC 20 μM (Δ^8^-THC 20 μM + Scratch). The gene transcription levels for each group were used to perform two different comparisons and inspect the transcriptomic difference between the groups considered.

**Figure 2:**
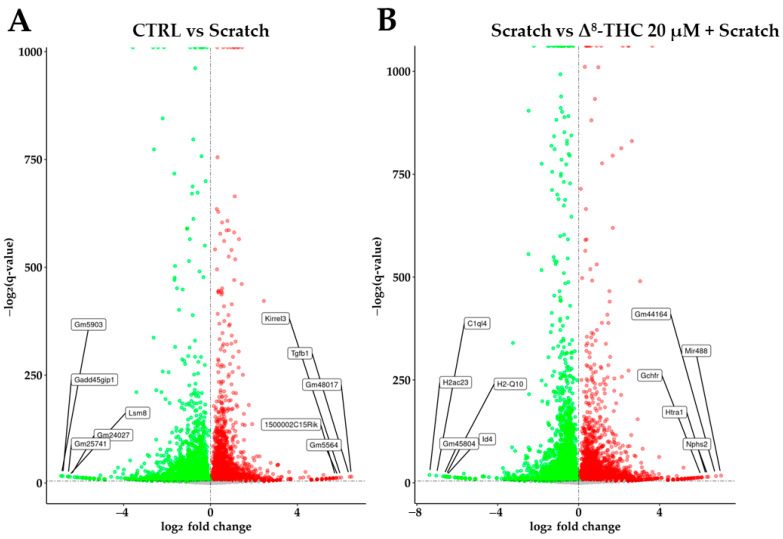
Volcano plot for CTRL vs. Scratch (**A**) and for Scratch vs. Δ^8^-THC 20 μM + Scratch (**B**). In the x axis is reported the log_2_ fold change so that upregulated genes are represented by red dots while downregulated genes by green dots. In the y axis is reported a score computed as the −log_2_(q-value). The labels are referred to the gene with mostly affected fold change.

The first comparison was performed between the CTRL and Scratch group (CTRL vs. Scratch) to underline the transcriptomic difference caused by the Scratch compared to CTRL group. The second comparison was performed between Scratch and Δ^8^-THC 20 μM + Scratch group (Scratch vs. Δ^8^-THC 20 μM + Scratch) to observe whether the pre-treatment acts on gene expressions avoiding the alteration induced by the scratch. For each gene, the statistical validation of the comparison was performed with the Wald test and the related p-value was corrected using Benjamini–Hochberg post hoc strategy to obtain the q-value. All the genes with a q-value < 0.05 were considered as differentially expressed genes (DEGs). In Table S1 are included all DEGs related to CTRL vs. Scratch. On the other hand, Table S2 collects all DEGs about Scratch vs. Δ^8^-THC 20 μM + Scratch. The volcano plots in Figure 2 highlight the distribution of the genes in the two comparisons. Thus, CTRL vs. Scratch (Figure 2A) resulted in 5924 DEGs among which 2897 downregulated and 3027 upregulated. Scratch vs. Δ^8^-THC 20 μM + Scratch (Figure 2B) resulted in 4830 DEGs among which 2375 downregulated and 2455 upregulated. For each volcano plot, we highlighted the mostly affected genes. Specifically, the five genes with higher fold change (both in up- and downregulation) were considered the mostly affected genes. In particular, *Gm48017*, *Tgfb1*, *Kirrel3*, *Gm5564* and *1500002C15Rik* are the most upregulated while *Gm5903*, *Gadd45gip1*, *Lsm8*, *Gm25741* and *Gm24027* are the most downregulated genes in the CTRL vs. Scratch experiment. On the other hand, the experiment Scratch vs. Δ^8^-THC 20 μM + Scratch has *Mir488*, *Gm44164*, *Nphs2*, *Htra1* and *Gchfr* as the most upregulated and *H2ac23*, *C1ql4*, *Gm45804*, *Id4* and *H2-Q10* as the most downregulated genes.

**Figure 3:**
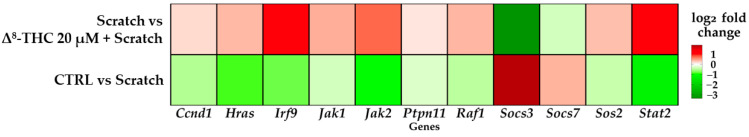
Heatmap of DEG fold change. Red palette is related to upregulated while green palette to downregulated DEGs. Fold change level refers to log_2_ fold change. All DEGs have opposite behavior among the two comparisons.

After a critical inspection of the pathway identified on KEGG and related to the biological context of our experiment, we put a focus on the “Jak-Stat signaling pathway” (mmu04630). The pathway counts on KEGG 171 genes. We found 33 out of 171 DEGs in CTRL vs. Scratch (with a ratio of 19%) and 38 in Scratch vs. Δ^8^-THC 20 μM + Scratch (with a ratio of 22%). Among them, 26 DEGs are in common between the two comparisons. Thus, we focused our attention on common DEGs with an opposite behavior that was involved in proliferation, cell-cycle progression and apoptosis, and they are plotted in Figure 3.

Noteworthy, among the 11 DEGs identified in the “Jak-Stat signaling pathway”, *Ccnd1*, *Hras*, *Irf9*, *Jak1*, *Jak2*, *Ptpn11*, *Raf1*, *Sos2* and *Stat2* are downregulated in CTRL vs. Scratch (more expressed in CTRL), while *Socs3* and *Socs7* are upregulated (more expressed in Scratch), as showed in the plot. Curiously, the nine DEGs downregulated in CTRL vs. Scratch are upregulated in Scratch vs. Δ^8^-THC 20 μM + Scratch while the two DEGs upregulated in CTRL vs. Scratch are downregulated Scratch vs. Δ^8^-THC 20 μM + Scratch. In order to have a complete landscape of aforementioned DEGs, in Table 1, we highlighted for each DEG the level of significance obtained by the analysis represented by a q-value for both comparisons.

**Figure 4:**
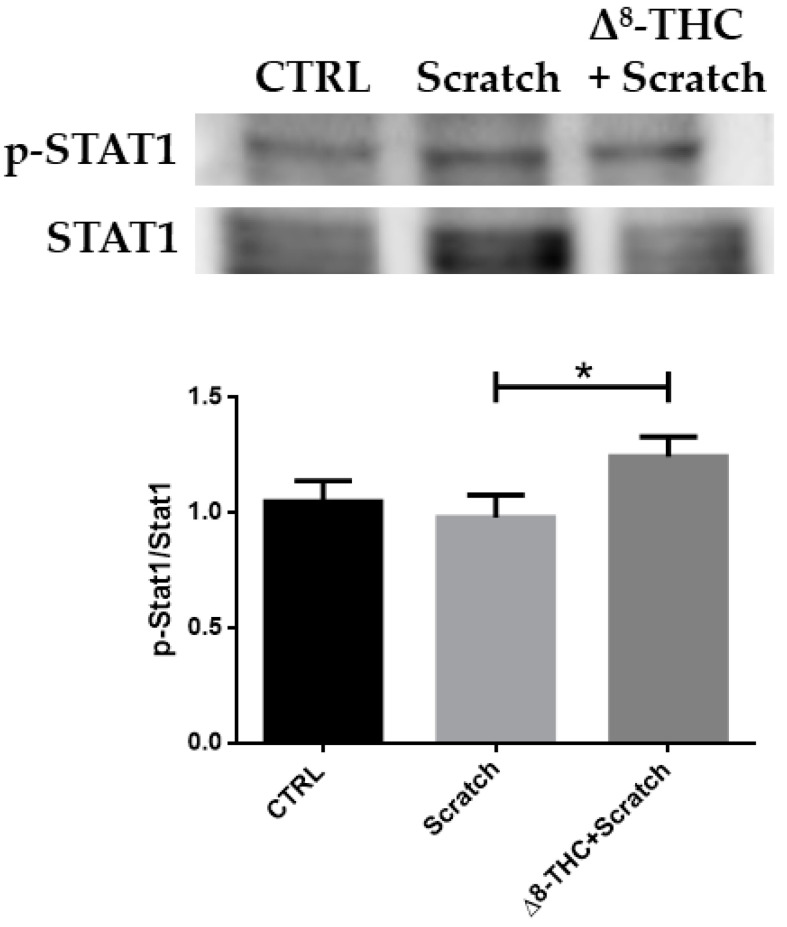
Western blot analysis of nuclear level of phosphorylated Stat1. Δ^8^-THC 20 μM increased the levels of phosphorylated Stat1. * *p* < 0.05.

In order to confirm the activation of the Jak-Stat pathway, we performed a Western blot analysis of Stat1. We observed the increased nuclear protein levels of phosphorylated Stat1 in the experimental group pre-treated with Δ^8^-THC 20 μM (Figure 4).

Since the Jak-Stat pathway also culminates in the “MAPK signaling pathway” (mmu04010) through Ras signaling, we also evaluated DEGs included in the aforementioned pathway (Table 2). Interestingly, only *Igf1r* was upregulated in the Ctrl vs. Scratch comparison. On the contrary, 13 upregulated DEGs were found in Scratch vs. Δ^8^-THC 20 μM + Scratch.

### 2.2. Gene Ontology Over-Representation Analysis

After KEGG pathways inspection, we also performed a gene ontology over-representation analysis to detect the significantly altered biological processes. In particular, we focused our attention on the terms enriched in both CTRL vs. Scratch and in Scratch vs. Δ^8^-THC 20 μM + Scratch comparisons.

**Figure 5:**
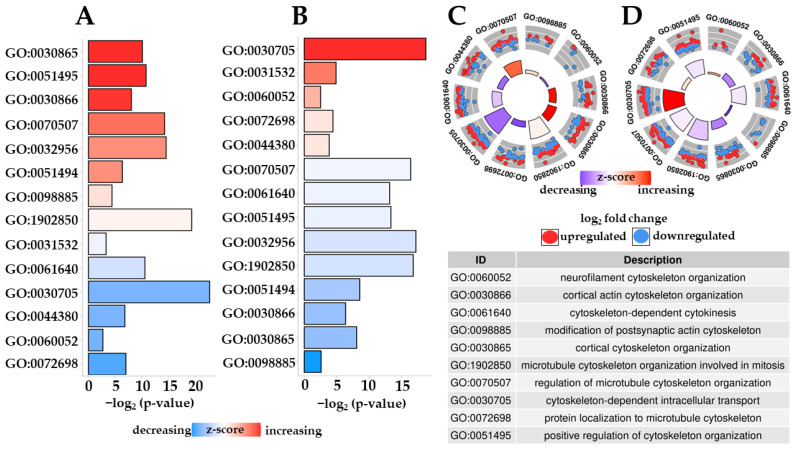
Bar plots (**A**,**B**) and circle plots (**C**,**D**) related to enriched biological process terms in both the comparisons. In the figure, *p*-value refers to adjusted *p*-value. Z-score is computed on the basis of the fold change for each gene belonging to the ontology. Panels A and C are related to CTRL vs. Scratch while panels B and D to Scratch vs. Δ^8^-THC 20 μM + Scratch. Panels C and D include the number of up- or downregulated DEGs involved in the specific term.

Additionally, due to the biological context of our experiment, our results were filtered out, choosing GO:0030705, GO:0030865, GO:0030866, GO:00331532, GO:0032956, GO:0044380, GO:0051494, GO:0051495, GO:0060052, GO:0061640, GO:0070507, GO:0072698, GO:0098885, and GO:1902850 that are involved in cytoskeletal reorganization. In Figure 5, we associated with each ontology the general deregulation of DEGs involved in the ontology itself. Only GO:0061640 is downregulated in both comparisons. All the other terms change its deregulation, from upregulation to downregulation or vice versa, in CTRL vs. Scratch against Scratch vs. Δ^8^-THC 20 μM + Scratch. Thus, in general, a change in the behavior is observed with Δ^8^-THC administration.

Among all the biological processes shown in Figure 5, cytoskeleton remodeling plays a crucial role in the case of cellular regeneration after an injury. For this reason, the ontology “neurofilament cytoskeleton organization” was investigated. For this biological process, we reported in Table 3 the fold change and q-value for each involved DEG.

## *3.* Discussion

In our experimental model, we used the differentiated motoneuron cell line NSC-34, which is suitable to replicate motoneuron diseases, to mimic the traumatic events [20,21,22,23]. In order to evaluate how Δ^8^-THC is able to modulate neuronal regeneration and cell death, we pre-treated the motoneuron cells with 20 µM Δ^8^-THC. The choice of the dose was based on previous studies. Indeed, in other previous experiments, we observed no cytotoxicity and the dose of 20 µM Δ^8^-THC was more suitable to reveal its effects [17].

After the treatment and the scratch injury assay, the transcriptome was analyzed to evaluate the Δ^8^-THC treatment’s effects on the pattern of gene expression of our motor neuron model.

As shown in Figure 3, the KEGG “Jak-Stat signaling pathway” reveals a very interesting behavior in our transcriptomic analysis. Indeed, this pathway includes *Ccnd1*, *Hras*, *Irf9*, *Jak1*, *Jak2*, *Ptpn11*, *Raf1*, *Sos2*, *Stat2*, *Socs3* and *Socs7* that change completely their fold change from up to down (or vice versa) in the experiments without Δ^8^-THC pre-treatment against Δ^8^-THC pre-treatment. The meaning of the behaviour is explainable by a strong response of the pathway after the injury events and, again, after the Δ^8^-THC treatment. Interestingly, the JAK-STAT signaling pathway can lead to the increase in the expression of growth factors, colony-stimulating factors (CSFs), hormones and interferons (IFNs) [24].

As the name suggests, the main trigger components of this pathway are JAK and STAT proteins. JAK1, JAK2, JAK3 and the Non-receptor tyrosine-protein kinase TYK2 (Tyk2) are different isomers of JAK proteins. Then, STAT1, STAT2, STAT3, STAT4, STAT5a, STAT5b and STAT6 belong to STATs protein family [25]. The pathway is active by homo-dimerization and hetero-dimerization of those proteins. Noteworthy, depending on the biological context, the pathway leads to different cellular responses such as cell differentiation, proliferation, migration, along with survival and apoptosis [26,27]. In our experimental model, we observed the downregulation of *Jak1*, *Jak2* and *Stat2* after the scratch experiment. Conversely, these DEGs are upregulated with Δ^8^-THC pre-treatment. These results suggest that the pathway is hindered by the injury. Simultaneously, Δ^8^-THC counteracts the traumatic event restoring the signalling. Western blot analysis evidenced the increased nuclear levels of phosphorylated Stat1 after the pre-treatment with Δ^8^-THC. Indeed, after Jak-dependent phosphorylation, Stat migrate into the nucleus. In parallel, we found the increase in signalling of the MAPK pathway with the pre-treatment and the upregulation of *Ccnd1*, so that we can speculate the reason for the activation of the cell-cycle progression. Indeed, *Ccnd1*, that encodes for the cyclin D1, has a pivot role in the regulation of the cell growth. STATs are known to activate cyclin D [28]. Moreover, RAS protein increases the expression of the cyclin D1, triggering the cell-cycle progression state in the G2 phase [29]. Additionally, we can observe the deregulation of *Irf9*. *Irf9* encodes for a regulatory factor of interferon and it, exactly as *Jak1*, *Jak2* and *Stat2*, is upregulated after scratch and downregulated with Δ^8^-THC pre-treatment.

It is interesting to note that, as proved by Platanitis, E. et al. [30], IRF9 complexes with STAT2 in the transcriptional activator interferon-stimulated gene factor 3 (ISGF3) [31,32]. Particularly, ISGF3 complex is involved in the activation of the signaling identified as the type-I IFNs JAK/STAT signaling towards the nucleus [33].

Additionally, our data show, along with the modulation in the activation of the pathway, its suppression in the signaling. Indeed, *Socs3* and *Socs7* are deregulated in opposition to the *Jak1*, *Jak2*, *Stat2* and *Irf9*. In detail, when *Jak1*, *Jak2*, *Stat2* and *Irf9* are upregulated, thus after the injury, *Socs3* and *Socs7* are downregulated and, vice versa, with pre-treatment Δ^8^-THC. Interestingly, *Soc3* has, respectively, the highest downregulation and upregulation among the inspected DEGs of the pathway. On the other hand, they could play a strong role in the drop of the signal in pre-treatment with Δ^8^-THC. Indeed, *Socs3* and *Socs7* encode for proteins of the SOCS family whose transcription and translation are regulated by STATs in a negative manner. Their synthesis stops the signal acting as negative feedback. Indeed, they hinder the substrate-binding socket of JAK proteins and block the transmission of the signals [34].

Particularly, different studies inspected the role of *Socs3* in neuronal regeneration. Smith, P.D. et al. [35] observed that the ablation of the *Socs3* gene supports the activation of adult retinal ganglion cells in JAK/STAT signaling, leading to axonal regeneration after optic nerve injury. *Socs3* deletion was also linked to a strong stimulus for the mouse corticospinal tract axonal regeneration [36]. In line with this study, the downregulation of *Socs3* induced by Δ^8^-THC pre-treatment suggests the predisposition of our cells to axon recovery after injury.

Also, proliferation and differentiation processes appear modulated in our experiment. In detail, *Ptpn11*, *Sos2*, *Hras* and *Raf1* are downregulated after scratch whereas they are upregulated with Δ^8^-THC pre-treatment. These proteins allow the initial crosslink of the pathway JAK-STAT with the MAPK signalling. The signalling induced by these genes seems, in this sense, reduced after the injury. Conversely the upregulation of these genes with the Δ^8^-THC treatment suggests an enhanced signalling. Looking at the expression of MAPK pathway genes following Ras and Raf, we observed the upregulation of the genes reported in Table 2 after the pre-treatment. Interestingly, we observed that the gene *Atf4*, encoding for a CREB family member, was upregulated. CREB proteins modulate different cellular processes such as cell proliferation, survival, and differentiation. In particular, in the nervous system, CREB family members are involved in neurogenesis, neuronal survival, neuronal plasticity, neurite outgrowth and neuroprotection [37]. Noteworthy, in the in vivo experiment of Liu, T. et al. [38], the MAPK signalling triggered by RAS protein supported an enhanced expression of the signalling after the injury in the spinal cord and it could be used to sustain the recovery from the damage. In this line, the modulation after the Δ^8^-THC treatment of the aforementioned DEGs supports the conclusion of the molecule activity in our model.

Due to the biological context of our experiment, we focus the attention on the mutual role of JAK-STAT and MAPK signaling pathway. Indeed, their deregulation could link the cytoskeletal rearrangement of our cells. Even if, to date, the circumstances are not clarified, several studies propose MAP kinases as drivers of the cytoskeletal rearrangement [39]. In particular, ATF4 was shown to have a role in regulating synapse formation and dendritic spine morphology [40]. Thus, we inspected the Gene Ontologies terms related to Biological Process terms that were enriched both after scratch and with Δ^8^-THC pre-treatment. From them, we selected the terms related to the cytoskeleton rearrangement that are plotted in Figure 5. It is very interesting to note that, generally speaking, most of the terms are downregulated after scratch and, conversely, upregulated with Δ^8^-THC pre-treatment. Noteworthy, the ontology “neurofilament cytoskeleton organization” (GO:0060052) is the ontology with the highest change in the deregulation of DEGs in the selected ontologies. After all, cytoskeletal reorganization is essential for recovering from traumatic injury [41].

For this reason, we then inspected the DEGs in this term that are collected in Table 3. Among them, *Cln8*, *Sod1* and *Vps54* are downregulated after the injury and upregulated after the treatment. Conversely, *Ina* is upregulated in the injury and downregulated in the treatment. Instead, *Ndel1* is upregulated in both comparisons. *Atf2* and *Atp8a2* are downregulated after scratch but are not differential with Δ^8^-THC pre-treatment. *Nefh* and *Nefl* are, respectively, up- and downregulated with pre-treatment Δ^8^-THC but not differential for the other comparison. In particular, *Nefh* and *Nefl* encode for the neurofilament-heavy and -light polypeptides, respectively. Neurofilaments are intermediate filaments of type IV located in the cytoplasm of the neurons [42,43]. They are usually involved in axonal transport, axon growth and maintenance [43,44]. Interestingly, Shaw, G. et al. [45] had already observed that, in serum, the hyperphosphorylated heavy neurofilament polypeptides can be used as biomarkers in axonal injury. Also, a pilot study of Hayakawa, K. et al. [46] highlighted a link between the level of that peptide and the severity of SCI in patients.

The highest change observed in the deregulation belongs to *Cln8* that is 10-fold different in the expression from scratch to Δ^8^-THC. Little is known but *Cln8* encodes for a transmembrane protein whose depletion was linked to lysosomes impairments in specific neurodegenerative disorders [47]. Ching, G.Y. et al. [48] generated transgenic mice to study the role of α-Internexin in neurodegeneration. α-Internexin protein is encoded by *Ina*. They discovered that a high level of this protein, as in our scratch comparison, leads to loss of neurons as a last consequence of neuronal dysfunction, progressive neurodegeneration. Interestingly, the Δ^8^-THC pre-treatment downregulates *Ina*.

The Cu,Zn-superoxide dismutase 1 is encoded by *Sod1*. Sugawara, T. et al. [49] showed that, after SCI, the overexpression of SOD1 hinders apoptosis in the in vivo experiments. In line with our data, Δ^8^-THC increases the expression of *Sod1* that was downregulated after scratch.

**Figure 6:**
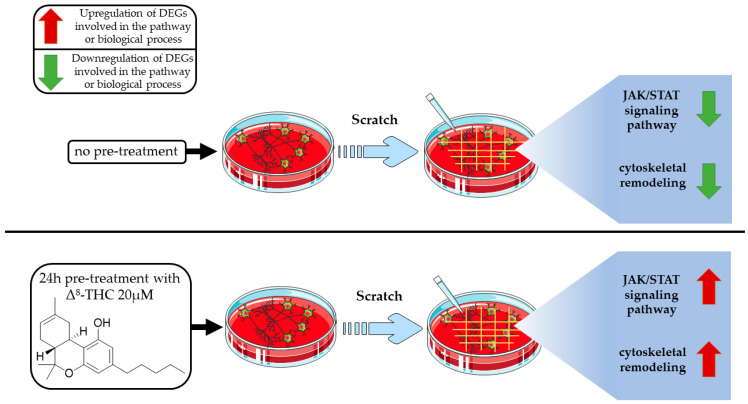
NSC-34 cells underwent different experimental treatments. In the upper frame, it the action of scratch injury technique is resumed on JAK-STAT signaling pathway and neurofilament cytoskeleton organization. Δ^8^-THC pre-treatment shows the ability to counteract the effects of scratch injury, as is illustrated in the lower frame.

As resumed in Figure 6, we show that Δ^8^-THC has the potential to counteract the effect of scratch injury both for JAK-STAT signalling pathway and cytoskeletal reorganization.

Even if this is the first study in which the Δ^8^-THC molecule was used to test its effect in a traumatic injury model, there are some considerations to point out. Indeed, NSC-34 cells are a specific cell population made of motoneurons. After a traumatic injury, other neuron cell types could be involved. In this sense, the in vitro model is not able to capture the whole complexity of an organism experienced by a traumatic injury. For this reason, our results should be validated in the following in vivo models.

## 4. Materials and Methods

### 4.1. Synthesis and Purification of Δ^8^-THC

CBD (200 mg, 0.636 mmol, 1eq) in Dichloromethane (DCM) (5 mL) was used as the base compound for the start of the synthesis of Δ^8^-THC. p-toluensulfonic acid (11 mg, 0.064 mmol, 0.1 eq) was added to the stirred solution and, for the following 6 h, the reaction was refluxed. After this step, Thin-Layer Chromatography (TLC) (Rf = 0.67, silica, petroleum ether-EtOAc 95:5) was assessed until the starting material was completely converted. It was later quenched with NaHCO_3_ s.s. and diluted with DCM. Washing with brine followed and then the organic phases were dried and evaporated. In order to obtain 182 mg (yield 91%) of Δ^8^-THC (1) as a brown oil, the residue underwent purification by Gravity Column Chromatography (GCC) on silica gel (pure petroleum ether to petroleum ether-EtOAc 9:1). A further step of purification was performed using JASCO Hichrom, 250 × 25 mm, silica UV−vis detector-2075 plus (silica, petroleum-ether-EtOAc gradient from 95:5 to 85:15), obtaining 150 mg of Δ^8^-THC (99% purity) as a brownish powder, whose structure has been identified in accordance with proton nuclear magnetic resonance (^1^H NMR) [17], using Bruker 400 spectrometers (Bruker^®^, Billerica, MA, USA), and the results were previously demonstrated [23]. The residues of solvent were identified as responsible for the chemical shifts (CDCl_3_: δH = 7.26). Low-pressure chromatography used Silica gel 60 (70–230 mesh) purchased from Macherey-Nagel (Düren, Germany). To assess the quality of the purifications, TLC on Merck 60 F254 (0.25 mm) plates was used, visualized by staining with 5% H_2_SO_4_ in EtOH and heating. Chemical reagents and solvents were from Aldrich (Darmstadt, Germany). HCPL JASCO Hichrom, 250 × 25 mm, silica UV−vis detector-2075 plus (Oklahoma, Japan).

### 4.2. NSC-34 Culture, Differentiation, and Treatment

The NSC-34 cell line was obtained from Cedarlane Corporation (Burlington, ON, Canada). NSC-34 cells were maintained in DMEM High Glucose supplemented with 10% Fetal Bovine Serum, 1% penicillin/streptomycin, and 1% L-Glutamine (Sigma-Aldrich, Merck KGaA, Darmstadt, Germany). For the treatment, 6-well plates were used to seed cells. A total of 24h after seeding, in order to induce cell differentiation, cells were incubated for 5 days with differentiation medium composed as follows: 1:1 DMEM/F-12 (Ham), 1% Fetal Bovine Serum, 1% L-Glutamine, 0.5% penicillin/streptomycin and 1 μM retinoic acid.

At the end of differentiation, cells were assigned to different experimental groups.

### 4.3. Scratch Injury and Δ^8^-THC Administration

Three different models were realized. In detail, the first model was made only from the differentiated NSC-34 cells and it was identified as the control (CTRL) group. The other two models were subjected to scratch injury. Firstly, the cells were incubated for 24 h with DMEM high glucose + 1% FBS + 1% Glutamine + 0.5% penicillin/streptomycin. Then, a 1 mL pipette tip was used to produce a mechanical injury on NSC-34 as previously described by Rajan, T.S. et al. [19]. In detail, the tip was moved 8 times. The first 4 movements were performed parallel to themselves. Each movement was made approximately 2 mm far from the previous one. Then, the plates were rotated 90 degrees and another 4 movements were performed as before, producing perpendicular movements to the previous ones. Additionally, one of the two model subjected to scratch injury was pretreated with 20 μM Δ^8^-THC for 24 h and it was identified as the Δ^8^-THC 20 μM + Scratch group. The scratch model without Δ^8^-THC pretreatment is referred to as the Scratch group.

### 4.4. RNA Isolation from Cells Pellet and cDNA Library Preparation

Total RNA was extracted using the Maxwell^®^ RSC simplyRNA Cells Kit (Promega, Madison, WI, USA) following the manufacturer’s instructions. Library preparation was carried out with the TruSeq RNA Exome protocol (Illumina, San Diego, CA, USA) [50]. The synthesis of the cDNA was made using the SuperScript II Reverse transcriptase (Invitrogen, Carlsbad, CA, USA). The cDNA adenylation and ligation at the 3′ end was made with adaptors. The library was amplified with PCR and clean-up took advantage of the AMPure XP beads (Beckman Coulter, Brea, CA, USA). The library was validated and the hybridization step was performed. To increase specificity against the regions that had to be captured, a second hybridization step was performed as before. The library was validated using the Tape Station. After the final step of normalization, the MiSeq Reagent Kit v3 by Illumina was used to sequence the library in single read mode on the MiSeq Instrument.

### 4.5. Bioinformatics Analysis

Resulting fastq files generated by the instrument were submitted to Sequence Read Archive databank identified with the BioProject PRJNA962508. In detail, the control group refers to experiment SRX20117387 (Transcriptomic of Mus Musculus without treatment) while the scratched group pre-treated with Δ^8^-THC refers to experiment SRX20117388 (Transcriptomic of Mus Musculus scratched with delta8-THC-20-microM pre-treatment). Additionally, we retrieved from the BioProject PRJNA791529 the already published scratched group without treatment with experiment ID SRX13480619 (Transcriptomic of Mus Musculus after scratch). In our study, we employed a range of software tools to evaluate the quality of sequencing reads, remove adapters and low-quality reads, align reads to a reference genome, and quantify gene expression. All the groups aforementioned were then analyzed in the same manner. FastQC version 0.11.9 was utilized to assess read quality (Babraham Institute, Cambridge, UK) [51] while Trimmomatic version 0.40-rc1 (Usadel Lab, Aachen, Germany) [52] was used to filter out adapter sequences and reads with low-quality scores. The resulting reads were then aligned to the vM28 genome from gencode using the STAR RNA-seq aligner version 2.7.10a_alpha_220207 (New York, NY, USA) [53], and transcript counts were obtained for each gene using HTSeq version 0.13.5 [54]. The differentially expressed genes were identified using DESeq2 library version 1.36.0 [55] in R version 4.2.0 (R Core Team). To drop the noise from the analysis, as suggested by the DESeq2 manual, we a priori removed all outlier genes with sum of the counts among all the samples lower than 10, keeping all the remaining genes. Then, because the samples were sequenced in different times, DESeq2 performed normalization among all the libraries and validated the results through the test statistics. In particular, we used the *estimateSizeFactors* and *estimateDispersionsGeneEst* functions to normalize both for length and depth. We corrected *p*-values using the Benjamini–Hochberg method with a threshold of 0.05 for the q-value. We further performed gene ontology (GO) enrichment analysis using the clusterProfiler version 4.4.3 [51] package in R, and inspected specific biological processes using the AmiGo2 database [56]. Additionally, we utilized the KEGG database [57] to gain insights into altered pathways in which the differentially expressed genes were involved. Our comprehensive bioinformatics pipeline enabled us to obtain robust gene expression data and to identify key biological pathways and processes that are involved in the system under investigation.

### 4.6. Protein Extraction and Western Blot

Cytoplasmic and nuclear protein extraction was performed with the NE-PER™ Nuclear and Cytoplasmic Extraction Reagents (Thermo Scientific™, Waltham, MA, USA) and their concentrations were evaluated using the Bradford assay (Bio-Rad, Hercules, CA, USA). Proteins were heated at 95 °C for 5 min, resolved by SDS-polyacrylamide gel electrophoresis (SDS-PAGE) and transferred onto a PVDF membrane (Immobilon–P, Millipore, Burlington, MA, USA). Membranes were blocked for 1 h at room temperature using 5% skim milk in TBS followed by incubation overnight at 4 °C with anti-Phospho-Stat1 antibody (1:1000; Cell Signaling Technology, Danvers, MA, USA) and anti-STAT1 (1:100; Abcam). After washing the membranes with TBS, they were incubated with HRP-conjugated anti-rabbit (1:1500; Santa Cruz Biotechnology Inc., Dallas, TX, USA). The relative expression of protein bands was visualized using an enhanced chemiluminescence system (Luminata Western HRP Substrates, Millipore, Burlington, MA, USA) and protein bands were acquired with the ChemiDoc™ XRS+ System (Bio-Rad, Hercules, CA, USA). Bands were quantified using the software ImageJ version 1.53t. 

### 4.7. Statistical Analysis

Statistical analysis was carried out with GraphPad Prism 9.0 software (GraphPad Software, La Jolla, CA, USA) using a one-way ANOVA test followed by Bonferroni post hoc test. A *p*-value ≤ 0.05 was considered statistically significant. The results are expressed by mean ± standard deviation (SD).

## 5. Conclusions

In this work, we showed the potential pharmacological role of Δ^8^-THC in a motoneuron cell line. NSC-34 cells that undergo traumatic scratch injury go towards cell death by dropping the signaling mediated by the JAK-STAT and MAP kinase pathways. Interestingly, the pre-treatment of NSC-34 cells with Δ^8^-THC before the injury ultimately leads to a recovery of the process reactivating these pathways. Additionally, the neurofilament cytoskeletal organization is the biological process related to cytoskeletal rearrangement with the highest differences from scratch against the Δ^8^-THC model. Our results in the motoneuron model suggest that Δ^8^-THC tries to revert the damage caused by trauma. In this line, further in vivo studies should confirm the role of cytoskeletal recovery through the JAK-STAT and MAP kinase pathway.

## Figures and Tables

**Table 1:** Genes involved in “Jak-Stat signaling pathway”.

Gene	CTRL vs. Scratch		Scratch vs. Δ^8^-THC 20 μM + Scratch	
	log_2_ Fold Change	q-Value	log_2_ Fold Change	q-Value
*Ccnd1*	−0.51	9.49 × 10^−26^	0.20	5.06 × 10^−5^
*Hras*	−0.88	1.16 × 10^−19^	0.37	3.29 × 10^−4^
*Irf9*	−0.79	6.00 × 10^−3^	1.25	4.80 × 10^−7^
*Jak1*	−0.32	7.47 × 10^−10^	0.42	8.36 × 10^−19^
*Jak2*	−1.07	4.29 × 10^−10^	0.75	9.20 × 10^−6^
*Ptpn11*	−0.27	4.55 × 10^−5^	0.14	2.74 × 10^−2^
*Raf1*	−0.46	2.51 × 10^−15^	0.38	7.62 × 10^−12^
*Socs3*	1.88	3.99 × 10^−2^	−3.35	4.67 × 10^−3^
*Socs7*	0.40	1.70 × 10^−3^	−0.32	5.00 × 10^−3^
*Sos2*	−0.40	3.25 × 10^−11^	0.34	2.90 × 10^−9^
*Stat2*	−1.25	3.62 × 10^−3^	1.28	1.50 × 10^−3^

For the 11 genes involved in Jak-Stat signaling pathway log_2_ fold change and q-value are reported in both comparisons. The q-value was computed using post hoc Benjamini–Hochberg correction. Values are rounded to the second decimal digit.

**Table 2:** Genes involved in “MAPK signaling pathway”.

Gene	Ctrl vs. Scratch		Scratch vs. DELTA8-20-Scratch	
	Fold_Change	q-Value	Fold_Change	q-Value
*Atf4*	-	>0.05	0.14	3.94 × 10^−6^
*Erbb3*	-	>0.05	0.74	6.75 × 10^−10^
*Grb2*	-	>0.05	0.27	3.76 × 10^−3^
*Hras*	-	>0.05	0.37	3.29 × 10^−4^
*Igf1r*	0.09	3.76 × 10^−2^	0.10	4.52 × 10^−3^
*Kras*	-	>0.05	0.34	1.72 × 10^−5^
*Map2k1*	-	>0.05	0.2	2.11 × 10^−7^
*Mknk1*	-	>0.05	0.75	3.39 × 10^−7^
*Ngfr*	-	>0.05	0.35	3.13 × 10^−3^
*Pdgfrb*	-	>0.05	1.42	9.52 × 10^−6^
*Raf1*	-	>0.05	0.38	7.62 × 10^−12^
*Rps6ka2*	-	>0.05	0.38	8.24 × 10^−17^
*Sos2*	-	>0.05	0.34	2.90 × 10^−9^

For the 13 genes involved in MAPK signaling pathway log_2_ fold change and q-value are reported in both comparisons. The q-value was computed using post hoc Benjamini–Hochberg correction. Values are rounded to the second decimal digit.

**Table 3:** DEGs involved in “neurofilament cytoskeleton organization” for both comparisons.

Gene	CTRL vs. Scratch		Scratch vs. Δ^8^-THC 20 μM + Scratch	
	Fold Change	q-Value	Fold Change	q-Value
*Atf2*	−0.11	1.40 × 10^−2^	-	-
*Atp8a2*	−3.12	9.86 × 10^−3^	-	-
*Cln8*	−5.14	1.89 × 10^−3^	5.90	1.94 × 10^−4^
*Ina*	0.44	3.75 × 10^−5^	−0.64	3.89 × 10^−11^
*Ndel1*	0.31	9.24 × 10^−6^	0.21	4.10 × 10^−4^
*Nefh*	-	-	0.20	4.51 × 10^−3^
*Nefl*	-	-	−0.32	2.28 × 10^−3^
*Sod1*	−1.30	5.83 × 10^−87^	1.35	2.06 × 10^−102^
*Vps54*	−0.41	2.86 × 10^−13^	0.13	2.60 × 10^−2^

For the 9 genes involved in neurofilament cytoskeleton organization term log_2_ fold change and q-value are reported in both comparisons. The q-value was computed using post hoc Benjamini–Hochberg correction. Values are rounded to the second decimal digit.

## Data Availability

The data presented in this study are openly available in the NCBI Sequence Read Archive at BioProject accession number PRJNA962508 and PRJNA791529 (https://www.ncbi.nlm.nih.gov/bioproject/?term=PRJNA791529).

## Supplementary Materials

The following supporting information can be downloaded at https://www.mdpi.com/article/10.3390/ph16091268/s1. Table S1: Full list of DEGs in CTRL vs. Scratch; Table S2: Full list of DEGs in Scratch vs. Δ^8^-THC 20 μM + Scratch.

## References

[1] Chi H., Chang H.Y., Sang T.K. (2018). Neuronal Cell Death Mechanisms in Major Neurodegenerative Diseases. Int. J. Mol. Sci..

[2] Shi Z., Yuan S., Shi L., Li J., Ning G., Kong X., Feng S. (2021). Programmed cell death in spinal cord injury pathogenesis and therapy. Cell Prolif..

[3] WHO Fact Sheet N°384. https://www.who.int/news-room/fact-sheets/detail/spinal-cord-injury.

[4] Eckert M.J., Martin M.J. (2017). Trauma: Spinal Cord Injury. Surg. Clin. N. Am..

[5] Wilson J.R., Cadotte D.W., Fehlings M.G. (2012). Clinical predictors of neurological outcome, functional status, and survival after traumatic spinal cord injury: A systematic review. J. Neurosurg. Spine SPI.

[6] Alizadeh A., Dyck S.M., Karimi-Abdolrezaee S. (2019). Traumatic Spinal Cord Injury: An Overview of Pathophysiology, Models and Acute Injury Mechanisms. Front. Neurol..

[7] Te Ao B., Brown P., Tobias M., Ameratunga S., Barker-Collo S., Theadom A., McPherson K., Starkey N., Dowell A., Jones K. (2014). Cost of traumatic brain injury in New Zealand: Evidence from a population-based study. Neurology.

[8] Ahuja C.S., Nori S., Tetreault L., Wilson J., Kwon B., Harrop J., Choi D., Fehlings M.G. (2017). Traumatic Spinal Cord Injury—Repair and Regeneration. Neurosurgery.

[9] Fernandez-Ruiz J., Moro M.A., Martinez-Orgado J. (2015). Cannabinoids in Neurodegenerative Disorders and Stroke/Brain Trauma: From Preclinical Models to Clinical Applications. Neurotherapeutics.

[10] Bhunia S., Kolishetti N., Arias A.Y., Vashist A., Nair M. (2022). Cannabidiol for neurodegenerative disorders: A comprehensive review. Front. Pharmacol..

[11] Tagen M., Klumpers L.E. (2022). Review of delta-8-tetrahydrocannabinol (Δ8-THC): Comparative pharmacology with Δ9-THC. Br. J. Pharmacol..

[12] Leas E.C. (2021). The Hemp Loophole: A Need to Clarify the Legality of Delta-8-THC and Other Hemp-Derived Tetrahydrocannabinol Compounds. Am. J. Public Health.

[13] LoParco C.R., Rossheim M.E., Walters S.T., Zhou Z., Olsson S., Sussman S.Y. (2023). Delta-8 tetrahydrocannabinol: A scoping review and commentary. Addiction.

[14] Bhatti F.I., Mowforth O.D., Butler M.B., Bhatti A.I., Adeeko S., Akhbari M., Dilworth R., Grodzinski B., Osunronbi T., Ottewell L. (2021). Systematic review of the impact of cannabinoids on neurobehavioral outcomes in preclinical models of traumatic and nontraumatic spinal cord injury. Spinal Cord.

[15] Nabata K.J., Tse E.K., Nightingale T.E., Lee A.H.X., Eng J.J., Querée M., Walter M., Krassioukov A.V. (2021). The Therapeutic Potential and Usage Patterns of Cannabinoids in People with Spinal Cord Injuries: A Systematic Review. Curr. Neuropharmacol..

[16] Anchesi I., Schepici G., Chiricosta L., Gugliandolo A., Salamone S., Caprioglio D., Pollastro F., Mazzon E. (2023). Delta(8)-THC Induces Up-Regulation of Glutamatergic Pathway Genes in Differentiated SH-SY5Y: A Transcriptomic Study. Int. J. Mol. Sci..

[17] Gugliandolo A., Blando S., Salamone S., Caprioglio D., Pollastro F., Mazzon E., Chiricosta L. (2023). Delta(8)-THC Protects against Amyloid Beta Toxicity Modulating ER Stress In Vitro: A Transcriptomic Analysis. Int. J. Mol. Sci..

[18] Cory G. (2011). Scratch-wound assay. Methods Mol. Biol..

[19] Rajan T.S., Diomede F., Bramanti P., Trubiani O., Mazzon E. (2017). Conditioned medium from human gingival mesenchymal stem cells protects motor-neuron-like NSC-34 cells against scratch-injury-induced cell death. Int. J. Immunopathol. Pharmacol..

[20] Citron B.A., Zhang S.X., Smirnova I.V., Festoff B.W. (1997). Apoptotic, injury-induced cell death in cultured mouse murine motor neurons. Neurosci. Lett..

[21] Payette D.J., Xie J., Shirwany N., Guo Q. (2008). Exacerbation of apoptosis of cortical neurons following traumatic brain injury in par-4 transgenic mice. Int. J. Clin. Exp. Pathol..

[22] Zhao Y., Luo P., Guo Q., Li S., Zhang L., Zhao M., Xu H., Yang Y., Poon W., Fei Z. (2012). Interactions between SIRT1 and MAPK/ERK regulate neuronal apoptosis induced by traumatic brain injury in vitro and in vivo. Exp. Neurol..

[23] Han Z., Chen F., Ge X., Tan J., Lei P., Zhang J. (2014). miR-21 alleviated apoptosis of cortical neurons through promoting PTEN-Akt signaling pathway in vitro after experimental traumatic brain injury. Brain Res..

[24] Darnell J.E. (1997). STATs and Gene Regulation. Science.

[25] Murray P.J. (2007). The JAK-STAT Signaling Pathway: Input and Output Integration. J. Immunol..

[26] Harrison D.A. (2012). The Jak/STAT pathway. Cold Spring Harb. Perspect. Biol..

[27] Ghoreschi K., Laurence A., O’Shea J.J. (2009). Janus kinases in immune cell signaling. Immunol. Rev..

[28] Pawlonka J., Rak B., Ambroziak U. (2021). The regulation of cyclin D promoters—Review. Cancer Treat. Res. Commun..

[29] Stacey D.W. (2003). Cyclin D1 serves as a cell cycle regulatory switch in actively proliferating cells. Curr. Opin. Cell Biol..

[30] Platanitis E., Demiroz D., Schneller A., Fischer K., Capelle C., Hartl M., Gossenreiter T., Muller M., Novatchkova M., Decker T. (2019). A molecular switch from STAT2-IRF9 to ISGF3 underlies interferon-induced gene transcription. Nat. Commun..

[31] Fu X.Y., Kessler D.S., Veals S.A., Levy D.E., Darnell J.E. (1990). ISGF3, the transcriptional activator induced by interferon alpha, consists of multiple interacting polypeptide chains. Proc. Natl. Acad. Sci. USA.

[32] Shuai K., Stark G.R., Kerr I.M., Darnell J.E. (1993). A single phosphotyrosine residue of Stat91 required for gene activation by interferon-gamma. Science.

[33] Ivashkiv L.B., Donlin L.T. (2014). Regulation of type I interferon responses. Nat. Rev. Immunol..

[34] Kershaw N.J., Murphy J.M., Liau N.P., Varghese L.N., Laktyushin A., Whitlock E.L., Lucet I.S., Nicola N.A., Babon J.J. (2013). SOCS3 binds specific receptor-JAK complexes to control cytokine signaling by direct kinase inhibition. Nat. Struct. Mol. Biol..

[35] Smith P.D., Sun F., Park K.K., Cai B., Wang C., Kuwako K., Martinez-Carrasco I., Connolly L., He Z. (2009). SOCS3 Deletion Promotes Optic Nerve Regeneration In Vivo. Neuron.

[36] Jin D., Liu Y., Sun F., Wang X., Liu X., He Z. (2015). Restoration of skilled locomotion by sprouting corticospinal axons induced by co-deletion of PTEN and SOCS3. Nat. Commun..

[37] Chowdhury M.A.R., An J., Jeong S. (2023). The Pleiotropic Face of CREB Family Transcription Factors. Mol. Cells.

[38] Liu T., Cao F.-j., Xu D.-d., Xu Y.-q., Feng S.-q. (2015). Upregulated Ras/Raf/ERK1/2 signaling pathway: A new hope in the repair of spinal cord injury. Neural Regen. Res..

[39] Bhattacharya A., Ghosh P., Prasad R., Ghosh A., Das K., Roy A., Mallik S., Sinha D.K., Sen P. (2020). MAP Kinase driven actomyosin rearrangement is a crucial regulator of monocyte to macrophage differentiation. Cell. Signal..

[40] Liu J., Pasini S., Shelanski M.L., Greene L.A. (2014). Activating transcription factor 4 (ATF4) modulates post-synaptic development and dendritic spine morphology. Front. Cell. Neurosci..

[41] Hoffman P.N., Lasek R.J. (1980). Axonal transport of the cytoskeleton in regenerating motor neurons: Constancy and change. Brain Res..

[42] Liu Q., Xie F., Siedlak S.L., Nunomura A., Honda K., Moreira P.I., Zhua X., Smith M.A., Perry G. (2004). Neurofilament proteins in neurodegenerative diseases. Cell. Mol. Life Sci. CMLS.

[43] Yuan A., Rao M.V., Nixon R.A. (2012). Neurofilaments at a glance. J. Cell Sci..

[44] Perrot R., Lonchampt P., Peterson A.C., Eyer J. (2007). Axonal neurofilaments control multiple fiber properties but do not influence structure or spacing of nodes of Ranvier. J. Neurosci..

[45] Shaw G., Yang C., Ellis R., Anderson K., Parker Mickle J., Scheff S., Pike B., Anderson D.K., Howland D.R. (2005). Hyperphosphorylated neurofilament NF-H is a serum biomarker of axonal injury. Biochem. Biophys. Res. Commun..

[46] Hayakawa K., Okazaki R., Ishii K., Ueno T., Izawa N., Tanaka Y., Toyooka S., Matsuoka N., Morioka K., Ohori Y. (2012). Phosphorylated neurofilament subunit NF-H as a biomarker for evaluating the severity of spinal cord injury patients, a pilot study. Spinal Cord.

[47] Rosenberg J.B., Chen A., Kaminsky S.M., Crystal R.G., Sondhi D. (2019). Advances in the Treatment of Neuronal Ceroid Lipofuscinosis. Expert Opin. Orphan Drugs.

[48] Ching G.Y., Chien C.-L., Flores R., Liem R.K.H. (1999). Overexpression of α-Internexin Causes Abnormal Neurofilamentous Accumulations and Motor Coordination Deficits in Transgenic Mice. J. Neurosci..

[49] Sugawara T., Lewén A., Gasche Y., Yu F., Chan P.H. (2002). Overexpression of SOD1 protects vulnerable motor neurons after spinal cord injury by attenuating mitochondrial cytochrome c release. FASEB J..

[50] Chiricosta L., Silvestro S., Pizzicannella J., Diomede F., Bramanti P., Trubiani O., Mazzon E. (2019). Transcriptomic Analysis of Stem Cells Treated with Moringin or Cannabidiol: Analogies and Differences in Inflammation Pathways. Int. J. Mol. Sci..

[51] Wu T., Hu E., Xu S., Chen M., Guo P., Dai Z., Feng T., Zhou L., Tang W., Zhan L. (2021). clusterProfiler 4.0: A universal enrichment tool for interpreting omics data. Innovation.

[52] Bolger A.M., Lohse M., Usadel B. (2014). Trimmomatic: A flexible trimmer for Illumina sequence data. Bioinformatics.

[53] Dobin A., Davis C.A., Schlesinger F., Drenkow J., Zaleski C., Jha S., Batut P., Chaisson M., Gingeras T.R. (2013). STAR: Ultrafast universal RNA-seq aligner. Bioinformatics.

[54] Anders S., Pyl P.T., Huber W. (2015). HTSeq—A Python framework to work with high-throughput sequencing data. Bioinformatics.

[55] Love M.I., Huber W., Anders S. (2014). Moderated estimation of fold change and dispersion for RNA-seq data with DESeq2. Genome Biol..

[56] Carbon S., Ireland A., Mungall C.J., Shu S., Marshall B., Lewis S., AmiGO Hub, Web Presence Working Group (2009). AmiGO: Online access to ontology and annotation data. Bioinformatics.

[57] Kanehisa M., Goto S. (2000). KEGG: Kyoto encyclopedia of genes and genomes. Nucleic Acids Res..

